# Bed Blocking by Hospitalized Patients Awaiting Admission to Intramural Aftercare: A Case Study on Transfer Coordination

**DOI:** 10.3390/healthcare13162038

**Published:** 2025-08-18

**Authors:** Josefine M. F. Janssens, Annelies van der Ham, Dirk Ruwaard, Frits van Merode

**Affiliations:** 1 Department of Health Services Research, Care and Public Health Research Institute (CAPHRI), Maastricht University, 6229 GT Maastricht, The Netherlands; 2 Amphia Ziekenhuis, 4818 CK Breda, The Netherlands; 3 Rhythm, 1014 AX Amsterdam, The Netherlands; 4 Care and Public Health Research Institute (CAPHRI), Maastricht University, 6200 MD Maastricht, The Netherlands; f.vanmerode@maastrichtuniversity.nl; 5 Maastricht University Medical Centre+, 6200 MD Maastricht, The Netherlands

**Keywords:** bed blocking, queuing system, patient flow, hospital, intramural care, capacity, coordination

## Abstract

*Background/Objectives*: Elderly patients who require aftercare in an intramural care (IMC) facility may contribute to “bed blocking,” which occurs when patients who are ready for discharge remain hospitalized longer than medically necessary. While most bed-blocking studies focus on capacity issues, this study also investigates the coordination process. In a regional hospital in the Netherlands, we examine the extent to which bed blocking occurs due to patients awaiting IMC, and how this issue can be characterized in terms of capacity and coordination challenges. *Methods*: The case study employs a mixed-methods approach, analyzing system data, documents, and interviews from the hospital, IMC organizations, and a health insurance provider. The location of each patient (organization and department) was collected and reconstructed to a patient path. All patient paths together formed a network enabling data analysis both on the level of patient paths as well as on the level of the networks as they developed through time. This gave insight into the complexity of the total network that has to be coordinated. *Results*: In 2023, 6% of the hospital capacity was occupied by patients awaiting IMC. Delays were observed at various coordination stages. Due to a lack of data on IMC bed capacity, we were unable to establish whether capacity limitations also contributed to bed blocking. *Conclusions*: The coordination system is complex and includes waiting times at each coordination stage, resulting in bed blocking. The absence of a centralized capacity overview, coupled with limited data, prevents decision-makers from identifying bed blocking arising from capacity shortages. Greater insight is needed to coordinate patient flow and determine the required slack capacity.

## 1. Introduction

Developed countries are facing the challenge of providing high-quality healthcare services for an aging population [1,2]. This challenge is particularly pressing as the number of individuals aged 65 and older continues to rise [1], while the health workforce is declining [3].

In Europe and North America, elderly people accounted for 18.7% of the population in 2022, a proportion expected to rise to 22% in 2030 and 26.9% in 2050 [4]. It is widely recognized that healthcare consumption increases with age, and that elderly patients consume a significant proportion of healthcare resources. Elderly individuals are often vulnerable and frequently require hospitalization [2], contributing to high occupancy levels in hospital wards. Hospital bed occupancy is influenced by stagnant patient flow, which occurs when patients who require admission to intramural aftercare (IMC) facilities, such as geriatric rehabilitation (GR), short-term residential care (STRC), hospices, and long-term care (LTC), remain in the hospital longer than medically necessary. This delayed discharge [1,5] has been referred to as bed blocking [2]. As the occupied hospital beds could have been used for other patients, bed blocking affects hospital accessibility and patient throughput. Furthermore, it is detrimental to the quality of care as the required rehabilitation for patients who remain hospitalized is delayed [2,6]. General insights about the number of patients with at least one bed blocking day globally are lacking, but some studies report that between 6% and 21% of patients experience delayed discharge [7,8,9].

Previous research indicates that capacity issues are a primary cause of bed blocking in hospitals, specifically the waiting times for alternative intramural accommodations [1,2,10] resulting from insufficient bed capacity in IMC facilities. This issue is particularly pronounced when patient arrivals are unpredictable or service times uncertain, creating challenges in consistently providing adequate capacity [11]. Additionally, patient preferences for specific IMC locations contribute to both bed blocking and the underutilization of IMC capacity: while beds may be available at one IMC facility, patients may prefer another location which is already at capacity.

Bed blocking is also linked to coordination issues. The elderly healthcare chain is complex and requires coordination of services. However, coordination beyond a basic queueing system is challenging, particularly when it involves various types of care across multiple healthcare organizations [12]. Moreover, the volume of demand and the case mix are often unstable. Inadequate design or coordination of the queueing system will inevitably result in patient delays [12].

A number of studies have employed queueing theory to analyze capacity planning and optimize bed and patient allocation to reduce waiting times, the length of waiting lists, and the associated costs [1,2,13,14,15,16]. The importance of coordination, however, tends to be overlooked. This study investigates whether bed blocking can primarily be attributed to capacity or coordination issues. The research question is: To what extent does bed blocking occur due to hospitalized patients awaiting IMC, and how can this issue be characterized in terms of capacity challenges and coordination issues?

## 2. Theory

Bed blocking refers to the phenomenon of delayed discharge, which occurs when patients are waiting for an available bed in IMC institutions [2]. Delayed discharge impacts hospital bed-occupancy levels. Some institutions report average occupancy rates [17], but these annual averages provide limited insight into occupancy variability, such as seasonal patterns or even hour-to-hour fluctuations [2]. According to Proudlove [16], the notion of a universally applicable occupancy rate, such as 85%, is a common misconception. The optimal occupancy rate is influenced by factors such as patient arrival rates, the service time of healthcare activities, and the variation in these variables due to fluctuating healthcare demand. Slack capacity is essential to accommodate unexpected variability in demand, and coordination of healthcare tasks should focus on matching healthcare demand with available capacity.

Effective coordination enables the timely completion of tasks and allows for a rapid response to problems arising within one organization or specialty, thereby minimizing the impact on other specialties or organizations in the chain [18]. For effective coordination, it is crucial that coordinators are empowered to make decisions and provided with timely information. This requires information transfer and collaborative decision-making across various positions and organizations within the chain [18]. However, complete coordination is rarely achievable in complex networks of organizations [12,18,19] and investing in information technology alone may not resolve all coordination challenges. Therefore, the design of organizations and healthcare systems should also focus on simplifying processes and structures to reduce complexity.

Strategies for complexity reduction aim to minimize the need for information processing without compromising performance. As the uncertainty of tasks and the frequency of exceptions increase, the capacity for information processing may become insufficient. In such cases, a redesign of the organization and/or organizational network should be considered [18]. Implementing an integrated control system, also known as a closed-loop system, enhances the control of complexity within the queueing system [19]. In a closed-loop system, information is not only continuously monitored and fed back into the system to adjust operations, ensuring that the system maintains the desired state, but the amount of information and coordination to do so is also controlled and kept within limits.

**Figure 1:**
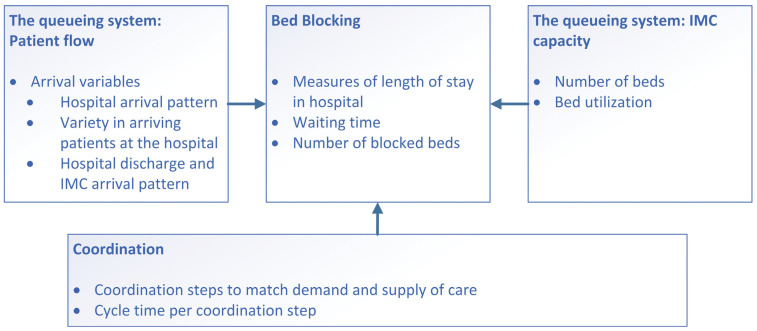
Conceptual model of this study.

Considering the above, in this study the following model is presented (Figure 1). Bed blocking is described using concepts from queueing theory [11] and coordination theory [18,20], with a focus on waiting time in the hospital and number of blocked beds, because this signals the extent of the bed blocking issue. The explanatory variables are therefore divided into three groups: (1) Patient flow characteristics of the queuing system (2) Capacity characteristics of the queuing system and (3) the coordination system. Patient flow is described in terms of arrival variables, including arrival rate, average inter-arrival time, and variation in arrival, because they affect the balance between demand and capacity in the healthcare setting. The service variables, such as length of hospital stay, waiting time, and number of blocked beds are also part of the total patient flow. The capacity variables include bed capacity and utilization, because they influence the patient flow and bed occupation in the hospital. Coordination is described in terms of coordination steps, their sequence, and cycle times. These variables are essential, as they can impact the patient flow, bed availability and can contribute to bed blocking.

## 3. Materials and Methods

### 3.1. Setting

This case study was conducted in the inter-organizational network in the eastern part of West Brabant, located in the southwestern Netherlands. The region has a population of over half a million inhabitants [20], with 21% of residents aged 65 years or older [21]. The population has an average socioeconomic status and relatively low perceived health compared to the overall population of the Netherlands [22].

The hospital primarily serving this region is Amphia Hospital, a top clinical teaching hospital in the city of Breda. In 2023, the hospital employed 5036 staff members, recorded 165,353 inpatient days, and generated a total revenue of €609 million [23,24]. Five IMC organizations participate in the network, Thebe, Avoord, Mijzo, Surplus, and De MARQ, collectively generating a total revenue of €419 million [25,26,27,28,29].

### 3.2. Study Design

This study uses the case-study methodology described by Yin [30]. The methodology was used in this study because it is suitable for theory development. While the functioning of queuing networks is theoretically well understood, the relationship between these networks and coordination remains insufficiently explored. Therefore, the argumentation was used to identify which variables describe ‘coordination’ in order to determine its effect on the overall healthcare system and, consequently, on bed blocking. This study focuses on both capacity allocation and the coordination of patient flows to shed light on the potential causes of bed blocking. We adopted a system-wide perspective, encompassing both the hospital and the IMC providers. Patients who went home after discharge or who died during hospitalization were excluded. A mixed-methods approach was used, incorporating the collection and analysis of data from various sources (quantitative data, documents, and interviews).

As shown in Figure 1, we first analyzed patient flow in terms of arrival and service variables (‘events’). Second, we examined bed blocking by measuring waiting time, defined as the period during which patients remained in the hospital after their medically ready date. Third, we analyzed IMC bed capacity and utilization. Finally, we investigated coordination in terms of coordination steps and the cycle time per step.

The way of describing events and processes follows the approach of considering patient paths as graphs (as in mathematical graph theory) where events are considered as nodes and transitions (from one location to another) as edges. Various patient paths are connected in a network (also a graph) by sharing nodes, thus by sharing capacity at a certain time. For further explanation and an overview of studies following this method see our paper [20]. For the methods the distinction was made in the conceptual model (Figure 1) between (1) Patient flow characteristics of the queuing system; (2) Capacity characteristics of the queuing system; (3) the Coordination system. This is important as according to the method we describe in paper [18] the distinction between (1) and (3) is crucial as the coordination system (3) puts constraints on the patient flow (1) and capacity use (2).

### 3.3. Data Collection and Analysis

System data were collected from three sources: the hospital information system (EPIC), the IMC organizations’ information systems and healthcare claims data from the largest healthcare insurer [31]. The IMC organizations use different information systems, but for this research a system called YSIS was mainly used. The data was first downloaded to Microsoft Excel and processed in order to have one standardized data set, which was uploaded to the Process Mining Toolkit (PMTK) of the Fraunhofen Institute. The eventlog of PMTK was used for further analysis. All data covered hospitalized patients who received aftercare at one of the IMCs. The hospital and IMC data spanned the period from the start of 2019 to the end of 2023, while the insurance data covered the start of 2019 to the end of 2022 (at the time of data collection, insurance data for 2023 were not yet available). The inclusion of 2019 data allowed for the examination of a pre-pandemic year, providing insights unaffected by the COVID-19 pandemic. Qualitative data were obtained from documents and seven semi-structured interviews. Figure 1 serves as the topic list in the interview guide.

#### 3.3.1. Queueing System: Patient Flow, Hospital, and IMC Arrival Patterns

Insurance data from the largest insurer in the region were used to analyze patient flow with the process mining software PMTK, covering approximately 50% of all patients served in the area. These data included healthcare claims with start and end dates for the various types of care provided to patients. The analysis focused on patients with at least one claim related to an inpatient hospital stay and at least one claim related to IMC.

To determine the number of patients in the flow, hospital data were retrieved from the hospital information system EPIC. Patients who had been hospitalized and subsequently received IMC were included regardless of insurance provider. For each hospital admission, the analysis included arrival and discharge dates, type of arrival (emergency or planned), medically ready date, and type of aftercare received.

Hospital system data were also used to analyze arrival and discharge patterns. For hospital arrivals, planned and emergency admissions were examined separately. The average number of daily hospital arrivals was calculated by dividing the total annual arrivals by the number of days from 2019 to 2023 inclusive.

Arrival patterns at IMC facilities were analyzed using the discharge dates of patients transitioning from the hospital to IMC. The hospital discharge date was considered the arrival date at the IMC facility. These discharges were then compared with arrival dates recorded in the IMC system data. In the systems of two out of five IMC organizations, the referring hospital was registered. In the hospital data, the specific IMC organization to which patients were transferred was recorded for only 2% of cases. Due to this limitation, total and daily average discharges to GR, STRC, hospice and LTC were analyzed.

To describe patient arrival patterns at the hospital, emergency and planned admissions were calculated separately, as differences in arrival numbers between these groups were expected. Additionally, medical specialization and diagnosis for each admission were extracted from the hospital system and incorporated into the main dataset for analyzing patient flow. This dataset was used to quantify the number of patients for whom different medical specializations placed IMC transfer orders.

#### 3.3.2. Queueing System: IMC Capacity and Utilization

IMC capacity includes the number of beds available for GR, STRC, hospice, and LTC. Both GR and STRC provide short-term care with the goal of helping elderly patients maintain or regain independence, although GR focuses on more complex rehabilitation needs following specialist hospital treatment. Hospice care is aimed at elderly patients with a life expectancy of up to three months. LTC is intended for elderly individuals who can no longer live independently.

There was no comprehensive overview of IMC bed capacity in the region, nor did any official documents detail the number of beds per organization. Therefore, a capacity overview was created for this study based on interviews and email correspondence with the managers of the IMC facilities. Additionally, the average bed utilization of the largest GR provider in the region was calculated. This calculation was based on system data from the IMC organization, where the total number of patient days (number of patients multiplied by length of stay) was divided by the total number of days in 2019 through 2023.

Analyzing bed utilization for the other IMC organizations and locations was not possible due to data limitations. The incompleteness of the data can be attributed to several factors, including insufficient data registration at these organizations, the absence of a unified system across IMC organizations due to mergers, and the lack of data specialists capable of extracting data from the existing systems.

#### 3.3.3. Bed Blocking: Length of Hospital Stay and Standard Deviation of Length of Stay

Hospital system data were used to analyze the length of hospital stay. To calculate the length of stay, admission date and time were subtracted from discharge date and time. The admission date refers to the date the patient was admitted to the hospital, while the discharge date refers to the date the patient left the hospital to transition to IMC. To calculate the average length of stay, the total length of stay for all patients was divided by the number of patients. The standard deviation of length of stay was also calculated.

#### 3.3.4. Bed Blocking: Number and Percentage of Bed-Blocking Days and Average Number of Blocked Beds Daily

The number of bed-blocking days was calculated by subtracting the medically ready date from the hospital discharge date for each patient. The medically ready date refers to the date on which the patient no longer requires medical intervention in the hospital. It is recorded by the medical specialist in the hospital information system.

The percentage of the length of stay during which a patient blocks a bed was calculated by dividing the number of bed-blocking days by the total number of days of the hospital stay. Finally, the average number of blocked beds per day was calculated by dividing the total number of bed-blocking days by the total number of days in 2019 through 2023.

#### 3.3.5. Coordination: Coordination Steps and Cycle Time

To identify the coordination steps, a process description of the triage process for patients requiring aftercare post-hospitalization was collected and analyzed. To understand when and how it is decided that a patient is transferred, roles, based on the constructed network of patient paths and capacity, were identified who oversee and/or perform the coordination of transfers. There were seven functions that fulfill these coordination roles. Of each function a person was identified for to participate in a semi-structured interview.

Four interviews took place within the hospital, with a transfer nurse, transfer manager, ward nurse, and medical specialist. A transfer nurse is responsible for arranging the transfer of the patients from hospital to IMC and can also be involved in the decision what type of IMC the patient needs. The transfer manager is the manager of the team of transfer nurses. As part of the transfer process to IMC, the medical specialist assesses the patient’s medical needs in the hospital, initiates the discharge planning, and is part of the coordination to ensure a safe and appropriate handover to IMC. A further three interviews were conducted with employees of the IMC organizations to gain a deeper understanding of the IMC process. The first interview involved a triage nurse, a healthcare worker who determines (in consultation with the transfer nurse) the type of aftercare required. The triage nurse also verifies whether there is available capacity within the IMC organization. The second interview was with the manager of the triage nurses at one of the IMC organizations. Finally, the third interview was with an elderly care physician, this is a physician who works primarily in intramural care settings, but is developing more as a transmural physician, and is specialized in the care of elderly patients with complex health problems.

The conducted interviews gave deeper insight into the coordination process and to construct an initial process outline. The time stamps of the coordination steps per patient were extracted from EPIC and were incorporated into the main dataset. This dataset was subsequently used to visualize the coordination path and their timing in a model created in Microsoft Visio. The process was as follows. Three moments in time were identified, namely hospital admission, medical ready date and hospital discharge. Coordination steps were positioned between these moments. Using the available time stamps, these coordination steps were plotted along the timeline to present the possible coordination paths. Since the dataset included time stamps for each coordination step per patient, it was possible to reconstruct the coordination ‘route’ for each individual case, as well as to determine the frequency of each distinct route. A list of the various coordination routes was made in Excel, and their frequency of occurrence was calculated. These routes were then further organized by type of care and type of hospital admission (emergency versus planned admission), as this information was also available at patient level. Using the time stamps, each route between two coordination steps was assigned a unique code, consisting of a letter and a number. The letter represents the coordination step at which the route ended, while the number indicates the timing relative to three key moments in the process: before hospital admission, between hospital admission and medically ready, and after medically ready. For example, all routes leading to coordination step 3 were assigned the letter A. The number 1 indicates that the coordination step occurred before hospital admission, number 2 signifies that it took place after hospital admission but before the patient was medically ready, and number 3 indicates that it happened after the medically ready date. Routes leading to hospital admission were coded as HA, followed by a letter to uniquely identify the route, while those leading to medically ready were coded as MR, followed by a letter.

The following records were excluded from the analysis:

- Records with medically ready date or a date for steps 3 to 6 later than the hospital discharge date, as these steps cannot occur after hospital discharge and were therefore considered registration errors;
- Records with a medically ready date before hospital admission, as a patient cannot be medically ready before admission;
- Records with an empty date field.

Note that only the specific coordination step was excluded from the analysis, not the entire coordination path. In some cases, the dates did not follow a numerical order from 1 to 7. In these instances, a flow path step is indicated with the same flow path number, but prefixed with a Z. For example, ZG2 represents flow path G2 in reverse. This occurred, for instance, when the transfer department began searching for an IMC bed for a patient (step 4) before the transfer order was placed (step 3). Time stamps were also used to calculate the cycle times of the coordination steps. Cycle time was defined as the duration it took to perform a step, including the time spent waiting for the step to begin (upon completion of the previous step). It was calculated by taking the time stamp of a coordination step minus the time stamp of the preceding coordination step. In some cases, this resulted in a negative cycle time, such as when the transfer order (step 3) was placed before the medically ready date (step 2).

Waiting time occurred when a coordination step was taken after the patient was medically ready. Due to typing errors, the dates for the coordination steps were not always recorded completely or accurately. A total of 13.7% of the time registrations for coordination steps 1 to 7 were blank, indicating that either the coordination step was not performed or not properly registered. Additionally, 22% of the patients had no medically ready date. To validate these data, several meetings were held with the team manager of the transfer nurses, after which the dates were adjusted accordingly.

## 4. Results

### 4.1. Queueing System: Patient Flow

**Figure 2:**
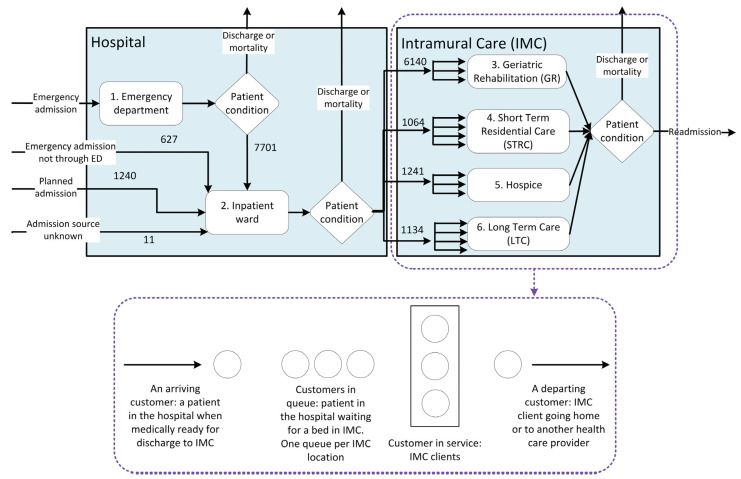
Number of patients transferred from the hospital to intramural care (IMC), 2019–2023.

Figure 2 presents the patient flow from the hospital to IMC facilities. From 2019 through 2023, a total of 143,996 patients were admitted to the hospital, encompassing all ages and diagnoses. Of these, 7% (9579 patients) were transferred to IMC after hospitalization, 92% of whom were aged 65 years or older. Among all patients, 87% arrived at the hospital via emergency admission, while 13% were planned admissions. The group of emergency patients has grown: “*The number of planned admissions has decreased over the past years.*” (Quote from interview, February 2024). Most emergency admissions involved general surgery, with 21% of total emergency admissions being transferred to IMC. Planned hospital admissions primarily involved orthopedic patients, with 38% of total planned admissions being transferred to IMC. Of the patients transferred to GR, 85% were emergency cases and 15% planned; for STRC and hospice, 90% were emergency and 10% planned; and for LTC, 94% were emergency and 6% planned (Appendix A). For emergency patients transferring to IMC, the arrival rate was 4.6 per day, with an inter-arrival time of 0.2 day between each arrival. For planned patients transferring to IMC, the arrival rate was 1 patient per day and inter-arrival time was 1 day between each arrival. Appendix A shows the arrival rates and inter-arrival times for each type of IMC.

Patients discharged to IMC after hospitalization received care from 15 different medical specializations within the hospital. Appendix A provides a breakdown of the number of patients per medical specialization and type of care. The medical specialist determines when a patient is medically ready for discharge based on their condition, without considering the available capacity at IMC facilities. Once patients are deemed ready for discharge and in need of IMC, they are placed in the IMC queue. As shown in Figure 2, 6140 patients (64%) transitioned to GR, 1064 patients (11%) to STRC, 1241 patients (13%) to hospice, and 1134 patients (12%) to LTC. The median number of hospitalized patients transferred to IMC was six per day (see Appendix B for further details).

### 4.2. Queueing System: IMC Bed Capacity

#### 4.2.1. Number of Beds

There are five IMC organizations in the region, encompassing 81 locations with a total of 93 to 96 departments. From 2019 through 2023, these facilities housed between 5035 and 5105 beds, with total capacity changing on 22 occasions due to the temporary or permanent opening or closing of locations and departments. Appendix C provides further details on the locations for each IMC type.

#### 4.2.2. Bed Utilization

Of the hospital patients transferred to GR, 57% were admitted to a single GR provider. As shown in Appendix D, the average bed utilization of this provider from 2019 through 2023 was 80% when only counting patients with GR status. However, GR beds were also occupied by patients awaiting LTC or those initially admitted as GR patients whose treatment was later reclassified as STRC. When accounting for all patients, average bed utilization was 89%.

Appendix D indicates that more beds were available than occupied. Although other IMC organizations were unable to provide bed-utilization data, interviewees suggested that not all beds were occupied at all times: “*We always have beds available, just not the ones the family or the patients wants.*” (Quote from interview, February 2025).

#### 4.2.3. Bed Blocking

A total of 37,055 bed-blocking days were recorded from 2019 through 2023. With a cumulative length of stay of 149,732 days for patients transitioning to IMC (Table 1), bed-blocking days accounted for 25% of the total hospital stay. This is also experienced by the ward nurses: “*Sometimes it takes quite a long time before patients can be transferred to a rehabilitation facility.*” (Quote from interview, February 2025). The median total length of stay for these patients was 12 days, while the median number of bed-blocking days was 3 (see Appendix E for details). At least one bed-blocking day was recorded for 62% of patients transferring to IMC after hospitalization (5967 patients).

On average, 20.3 beds were occupied by patients awaiting IMC. The number of bed-blocking days, the percentage of bed-blocking days relative to the total hospital stay, and the average number of daily blocked beds all increased from 2019 to 2023, except during the primary COVID-19 years, 2020 and 2021. The average number of daily blocked beds was 20.2 in 2019, 21.6 in 2022, and 29.7 in 2023. The percentage of bed-blocking days relative to the total hospital stay was 24% in 2019, 26% in 2022, and 33% in 2023. Appendix F provides a detailed breakdown of bed-blocking days and related metrics per year.

#### 4.2.4. Coordination: Coordination Steps

Seven steps were involved in coordinating the transfer of patients from the hospital to IMC:

1. Hospital admission: The medical specialist decides to admit the patient to the hospital.
2. Medically ready: The medical specialist determines that the patient has completed medical treatment and is ready for discharge.
3. Order to transfer: The ward nurse submits a transfer order to the transfer department for IMC aftercare.
4. Open the order: The transfer nurse reviews the transfer order, examines the patient’s case, and searches for a suitable, available bed at an IMC facility.
5. Registration at IMC facility: The transfer nurse registers the patient at the selected IMC facility.
6. Confirmation from IMC facility: The IMC facility confirms the patient’s transfer, although the exact transfer date remains unspecified.
7. Hospital discharge: The patient is transferred to the IMC facility.

**Figure 3:**
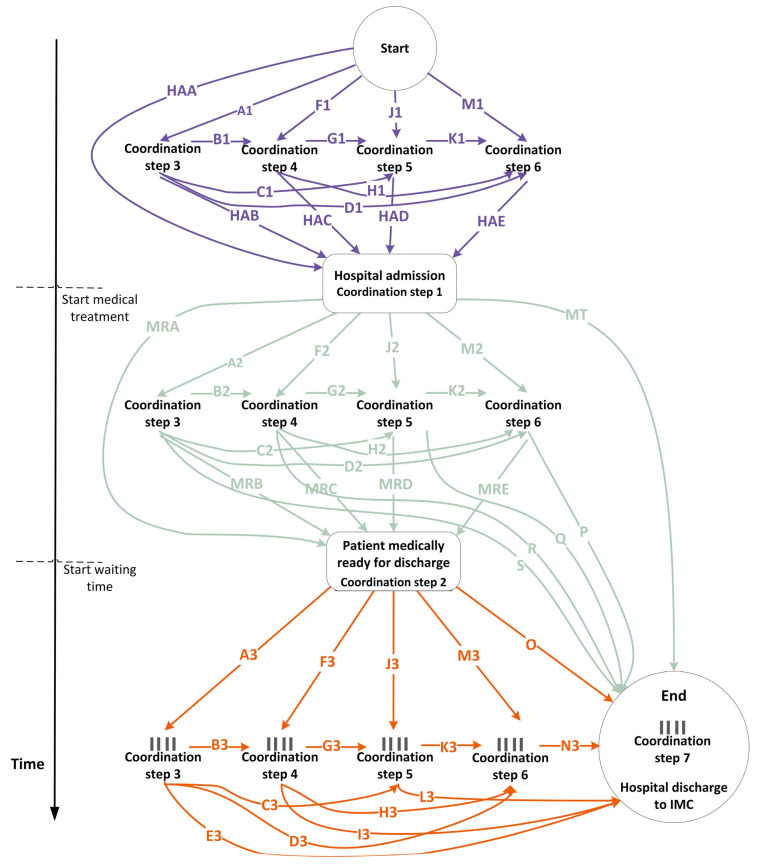
Coordination steps (**1**) before hospital admission, (**2**) after hospital admission but before medically ready, and (**3**) after medically ready but before hospital discharge. 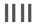 = potential queue.

Steps 1, 2, and 7 directly concerned the patient’s medical treatment. The other coordination steps took place at various times and in different sequences, depending on whether the case involved a planned (13%) or an emergency (87%) patient. For planned patients, steps 3 to 6 occurred either before or after hospital admission and before or after the medically ready date. For emergency patients, coordination steps 3 to 6 occurred either before or after the medically ready date (step 2), but always after hospital admission (step 1). The coordination steps are illustrated in the process flow in Appendix G and Appendix H. Patients experienced delays when steps 3 to 6 and/or hospital discharge (step 7) occurred after the patient was medically ready for discharge. All coordination steps and their sequence are depicted in Figure 3.

Finally, patients have the option to indicate their preferred IMC organization and location. The transfer nurse then checks bed availability by contacting the IMC facility by email or phone. If no bed is available at the preferred location, the patient is registered at an alternative IMC facility. As a result, from coordination step 6, the process diverges into multiple queues (i.e., queues for each bed type) at each IMC location. The total waiting time, defined as the time spent on coordination steps 3 to 7 after the medically ready date, represents the number of bed-blocking days.

Figure 3 illustrates the coordination process, with each line assigned a unique code. Route HAA represents patients for whom no coordination occurred before hospital admission. For patients following route MRA, there was no coordination between hospital admission and the medically ready date. Route MT indicates that medical treatment occurred, but the medically ready date was not recorded. These variations result in eight distinct scenarios for the execution of coordination steps 3 to 6:

1. Steps 3 to 6 are executed before hospital admission.
2. Steps 3 to 6 are executed after hospital admission and before the medically ready date.
3. Steps 3 to 6 are executed partly before hospital admission and partly after hospital admission, but before the medically ready date.
4. Steps 3 to 6 are executed after the medically ready date.
5. Steps 3 to 6 are executed after hospital admission, with some steps occurring before the medically ready date and others after.
6. Steps 3 to 6 are executed partly before hospital admission, and partly after the medically ready date.
7. Steps 3 to 6 are executed partly before hospital admission, partly after hospital admission, and partly before and after the medically ready date.
8. No coordination steps are executed. This applies only to route HAA-MT, where none of steps 3 to 6 were recorded.

A total of 155 different routes were identified (Appendix I, Appendix J, Appendix K, Appendix L, Appendix M, Appendix N and Appendix O). Of all cases, 5254 (55%) followed the top 5 routes, which are as follows:

1. HAA-A2-B2-G2-MRD-M3-N3 1640 cases (17%) category: 5
2. HAA-A2-B2-G2-K2-MRE-O  1570 cases (16%) category: 5
3. HAA-A2-MRB-F3-G3-K3-N3   813 cases (8%)  category: 5
4. HAA-MRA-A3-B3-G3-K3-N3  680 cases (7%)  category: 4
5. HAA-A2-B2-G2-K2-P       551 cases (6%)  category: 2

To illustrate, the order of coordination steps for route HAA-A2-B2-G2-MRD-M3-N3 is as follows: step 1: hospital admission (route HAA)—step 3: order to transfer (route A2)—step 4: open the order (route B2)—step 5: registration at IMC facility (route G2)—step 2: medically ready (route MRD)—step 6: confirmation from IMC facility (route M3)—step 7: hospital discharge (route N3).

For 60 routes, only one case was recorded (1%). For 77 routes, between 2 and 94 cases were recorded per route, totaling 1253 cases (13%). For 13 routes, between 100 and 499 cases were recorded per route, amounting to 3012 cases (31%).

In 7340 cases (77%), at least one coordination step between steps 3 and 6 occurred after the medically ready date, resulting in bed blocking. “*Ideally, discharge planning should start at the time of admission, but in practice, it often starts quite late.*” (Quote from interview, January 2024). In 82 cases (1%), the transfer coordination steps took place before hospital admission. In 2006 cases (21%), all transfer coordination steps occurred before the medically ready date, primarily involving neurology patients. For 21% of neurology patients, coordination was completed before the medically ready date. Yet, neurology also includes the largest percentage of cases in which all coordination steps took place after the medically ready date (scenario 4). The transfer orders for neurology patients are not handled by the transfer department but by a transfer nurse dedicated to neurology. For neurology patients requiring IMC, 71% had been involved in a cerebrovascular accident (CVA). Coordination was completed for 28% of these CVA patients before the medically ready date, and for 72% thereafter.

Although coordination for most CVA patients occurred after the medically ready date, the average number of bed-blocking days for these patients was five days. This is the same as the average number of bed-blocking days for GR (emergency and planned patients), as patients with CVA are given priority at GR facilities.

#### 4.2.5. Coordination: Cycle Time and Waiting Time

The median cycle time for coordination step 2, which represents the waiting time until medically ready and the process time for medically ready, was 7 days for planned care and 11 days for emergency care (Appendix P and Appendix Q). The cycle time for step 3 was -4 days for planned care and -3 days for emergency care. This indicates that the order to transfer (step 3) was typically placed before the medically ready date, in line with the policy of sending the transfer order as early as possible, preferably before the medically ready date.

For each subsequent coordination step (steps 3 to 6), the median cycle time was zero to one day for planned care and zero to two days for emergency care. The final step, hospital discharge (step 7), had a median cycle time of three days for planned care and two days for emergency care. Appendix P and Appendix Q provide additional details on the mean, median, and standard deviation for each type of care.

**Figure 4:**
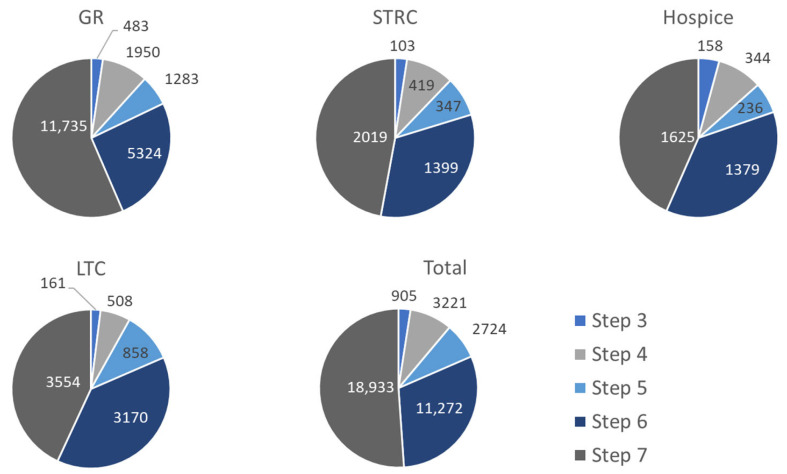
Bed-blocking days per coordination step for hospitalized patients transferring to IMC, 2019–2023. GR = geriatric rehabilitation, STRC = short-term residential care, LTC = long-term care.

Of the total bed-blocking days, 51% were attributed to patients either waiting for or undergoing step 7 (including patients waiting for discharge). This also encompasses waiting time for discharge when no coordination step was registered before step 7. For coordination step 6, 25% of the data registration was missing, which led to the addition of waiting time to the waiting period for step 7. Despite the missing data, step 6 accounted for 30% of the bed-blocking days. The waiting times for each coordination step are shown in Figure 4.

## 5. Discussion

In this section the following topics are discussed: a brief summary of the research findings, the complexity and instability of the coordination system, patients waiting for the blocked beds and the associated ethical dilemma, suggestions for controlling the healthcare chain, limitations of the study and recommendations for future research.

This study investigated the extent to which bed blocking occurred in a regional healthcare network due to patients awaiting IMC, and how this issue can be characterized in terms of IMC capacity and coordination processes. We found that 6% of hospital beds in 2023 were occupied by patients awaiting IMC and bed blocking increases, from 2019 (20.2 average blocked beds daily) to 2023 (29.7 average blocked beds daily), which has consequences relevant to practice). This can affect the available capacity for patients needing hospital care. Bed blocking was primarily caused by delays in coordination. Due to a lack of data, we cannot confirm whether it was also attributable to a capacity shortage at IMC facilities.

The 155 different coordination routes and the high standard deviation of length of stay and number of bed-blocking days suggest a lack of stability in the coordination system, resulting in bed blocking. The regional healthcare network exhibits typical characteristics of an open-loop system. The system is inadequately controlled because there is no provision for patients at the end of the chain, and the organizations themselves have contributed to its complexity. A lack of efficient coordination can lead to significant variation in employee workload throughout the week and even on a daily basis [12,19]. This variability can result in a permanently unstable system, development of queues and an uncontrolled amount of coordination effort.

This study helps shed light on the phenomenon of bed blocking by closely examining coordination, a perspective often neglected in existing research. We show that coordination is complex and involves waiting times at every step. Several stakeholders influence capacity utilization and patient flow, yet there is no centralized, reliable overview of total IMC bed capacity and availability. The absence of such an overview creates uncertainty in coordination and contributes to inefficiency. As indicated by Galbraith [32], the greater the uncertainty in the coordination task, the more difficult it is to plan capacity and the more information is required.

One would need to consider to what extent bed blocking is truly problematic. Although this study utilizes commonly accepted bed-blocking definitions from the literature [1,2], it remains unclear whether beds are genuinely blocked. Bed blocking would be problematic if there were patients awaiting hospital admission but no available beds. However, if a patient is waiting in the hospital for an IMC bed, and no other patients are waiting for the occupied hospital bed, then from a capacity perspective, bed blocking need not be problematic. Preventing bed blocking could lead to choices being made regarding patients’ transfers, which could raise ethical considerations with regard to which patient is prioritized over others. For example, occupied intensive care beds may hinder the admission of patients who need intensive care after surgery.

Furthermore, slack capacity should be taken into account when controlling the healthcare chain. For any coordination system, information on these variables is essential. Without such data, it remains unclear whether the coordination system is functioning effectively, or even how well it could theoretically perform.

The limitations of the coordination system can also be seen as limitations of the research itself. Although the extent to which bed blocking is truly problematic remains unknown, as the data on patients waiting for these beds were not available. It is undoubtedly a relevant issue in the Dutch healthcare system, where existing waiting lists and access time standards frequently go unmet. Furthermore, the aging population in developed countries and the decline in the healthcare workforce [1,2,3] place additional pressure on timely access to healthcare.

The constructed network of patient paths allowed to derive coordination/transfer decisions. However, it might be possible that sometimes coordination is informal and not registered. That could mean that the true extent of bed blocking is larger than observed in this study. The absence of data on which IMC organization patients were transferred to also prevented us from analyzing potential differences in how each organization allocates capacity to hospitalized patients.

Further, the IMC organizations investigated lacked a clear and reliable overview of bed capacity, the number of patients in their care, and their length of stay. This data deficit not only limits the research itself, but—more importantly—also complicates the effective management and control of the regional healthcare system. Insight into bed capacity is a precondition for managing bed capacity regionally, enabling closed-loop management. Future research could shed light on whether our findings are specific to this region or more generally applicable. It should focus on gaining a deeper understanding of information processing and decision-making within coordination processes. Additionally, future studies should explore how coordination can contribute to the development of a closed-loop system, where slack capacity is integrated into decision-making to ensure a smooth patient flow. This study shows that coordination plays a crucial role in bed blocking. This finding heightened the urgency in the region to establish a more centralized overview of total IMC capacity. The region has since invested in a system in which IMC organizations register their capacity. In line with Galbraith [32] information-processing capacity has been enhanced by reducing the division of labor and creating a small, autonomous group to perform coordination tasks. In late 2024, the coordination of short-term IMC was centralized, with all triage nurses from different IMC organizations now working from a single location to coordinate patient transfers. This can help future research on whether bed blocking is also attributed to capacity shortage at IMC facilities. This also marks the first step in transitioning to a closed-loop system. Future steps should involve incorporating available IMC capacity into decision-making regarding hospital admission, treatment, and discharge. Developing and implementing integrated IT systems for coordination places significant demands on resources and may require organizational restructuring. Since closed-loop coordination does not necessarily depend on centralization, an alternative strategy could involve accepting decentralized patient flow management, but promoting flexibility in capacity.

We think the method is applicable to other regions as well. A major problem in this region is the effort to obtain the data. Another reason is the use of various software systems and lack of data standardization. These problems may also occur in other regions. This is not a principle obstacle for replicability. For regular quality improvement programs this however is a major problem and for standardization of data and IT systems, interoperability is necessary.

Further research is needed to determine the best IT and data structure at a regional level. But this should be preceded by studying how the coordination of patient flows and capacity can be optimized. This is especially urgent as we observe that sometimes scarce capacity is not used as the coordination of patient flows and capacity assignment is ineffective.

## 6. Conclusions

The main conclusion of this study is that bed blocking in the hospital under investigation should be viewed primarily as a symptom of a problematic coordination system. There is no comprehensive overview of the capacity of the entire regional healthcare system, and it is not being effectively controlled. To achieve such control, the system would need to be designed with a clear understanding of the required bed capacity, based on the number of patients, their arrival patterns, the necessary slack bed capacity, and the acceptable service levels.

## Figures and Tables

**Table 1:** Hospital and bed blocking frequency, 2019–2023.

	Year 2019–2023	GR	STRC	Hospice	LTC	Total
Medically ready date vs. date of discharge (waiting time)	No. patients and % of total of type of care with at least 1 bed-blocking day	3994 65%	604 57%	679 55%	690 61%	5967 62%
	No. patients and % of total of type of care discharged on medically ready date	795 13%	208 20%	172 14%	198 17%	1373 14%
	No. patients and % of total of type of care with medically ready date post-discharge or missing	1351 22%	252 23%	390 31%	246 22%	2239 23%
	Total no. patients	6140	1064	1241	1134	9579
Number of bed-blocking days	Total no. bed-blocking days	20,775	4287	3742	8251	37,055
Bed blocking vs. length of stay Bed occupation	Total no. days in hospital	91,984	16,322	17,142	24,285	149,732
	% of bed-blocking days out of total length of stay in hospital	23%	26%	22%	34%	25%
	Average no. occupied beds daily	11.4	2.3	2.0	4.5	20.3

Note: GR = geriatric rehabilitation, STRC = short-term residential care, LTC = long-term care.

## Data Availability

Data are not available (as stipulated in the DTA). Further inquiries can be directed to the corresponding author(s).

## Abbreviations

The following abbreviations are used in this manuscript:

IMC	Intramural care
GR	Geriatric rehabilitation
STRC	Short-term residential care
LTC	Long-term care
CVA	Cerebrovascular accident

## Appendix A. Variation in Case Mix

**Year 2019–2023**	**GR**	**STRC**	**Hospice**	**LTC**	**Total**
**No. planned arrivals** **% of total arrivals**	938 15%	108 10%	127 10%	67 6%	1240 13%
**No. emergency arrivals** **% of total arrivals**	5193 85%	956 90%	1113 90%	1066 94%	8328 87%
**Average arrival rate/day: planned patients (arrive on Mon–Fri)**	0.7	0.08	0.1	0.05	1
**Average arrival rate/day: emergency patients (arrive on Mon–Sun)**	2.8	0.5	0.6	0.6	4.6
**Inter-arrival time/day (24 h): planned patients**	1.4	12.5	10	20	1
**Inter-arrival time/day (24 h): emergency patients**	0.4	2	1.7	1.7	0.2
**M length of stay (days)**	15	15	14	21	16
**SD in length of stay**	13.1	13.6	10.0	15.4	13.3
**M no. patients discharged from hospital to IMC/day**	3.4	0.6	0.7	0.6	5.2
**SD in no. patients discharged from hospital to IMC/day ***	2.5	0.9	0.8	0.9	3.5
**No. medical specializations**	15	14	13	12	15
Note. M = mean; SD = standard deviation; GR = geriatric rehabilitation; STRC = short-term residential care; LTC = long-term care; IMC = intramural care. * Calculated using all dates from 2019 through 2023 and the no. discharges/date, incl. weekends and national holidays (where discharged patients = 0).					

## Appendix B. Mean, Median and Standard Deviation of Number of Patients Discharged from Hospital to IMC per Day

**Year 2019–2023**	**GR**	**STRC**	**Hospice**	**LTC**	**Total**
**M no. patients discharged from hospital to IMC/day**	4.1	1.4	1.4	1.5	5.9
**Mdn no. patients discharged from hospital to IMC/day**	4	1	1	1	6
**SD of no. patients discharged from hospital to IMC/day**	2.1	0.76	0.6	0.8	3.1
Note. M = mean; Mdn = median; SD = standard deviation; GR = geriatric rehabilitation; STRC = short-term residential care; LTC = long-term care; IMC = intramural care.					

## Appendix C. IMC Bed Capacity

This table shows the bed capacity, number of providers, and locations for each type of IMC (minimum and maximum numbers of beds/locations/organizations).

**Year 2019–2023**	**GR**	**GR/STRC**	**STRC**	**Hospice**	**LTC**	**LTC/STRC**	**IMC Total**
**No. beds**	8	235–270	58–89	33	4597–4715	17–57	5035–5105
**No. locations/departments**	1	7–10	11–12	5	65–66	2–4	93–96
**No. organizations**	1	2–3	3–4	3	4	1	5
**No. times capacity changed**	5	3	3	0	5	6	22
Note. GR = geriatric rehabilitation; STRC = short-term residential care; LTC = long-term care; IMC = intramural care.							

## Appendix D. Bed Utilization at the Largest GR Provider 2019–2023

The figure below shows the total number of GR patients (orange) and the combined total of GR, STRC, and LTC patients (blue). The gray line represents the bed capacity.

## Appendix E. Length of Hospital Stay and Bed-Blocking in Days

**Year 2019–2023**	**GR**	**STRC**	**Hospice**	**LTC**	**Total**
**M length of stay (days)**	15.0	15.3	13.8	21.4	15.6
**Mdn length of stay**	11.5	11.1	11.2	17.6	12.0
**SD of length of stay**	13.1	13.6	10.0	15.4	13.3
**M bed-blocking days**	4.1	4.6	3.9	8.4	4.7
**Mdn of bed-blocking days**	3.0	3.0	3.0	6.0	3.0
**SD of bed-blocking days**	11.5	25.3	13.5	21.1	13.8
Note. M = mean; Mdn = median; SD = standard deviation; GR = geriatric rehabilitation; STRC = short-term residential care; LTC = long-term care.					

## Appendix F. Number of Bed-Blocking Days per Year

	**Year**	**GR**	**STRC**	**Hospice**	**LTC**	**Total**
**No. patients and % of total per type of care with at least 1 bed blocking day**	2019	825 (64%)	91 (62%)	154 (66%)	129 (72%)	1199 (66%)
	2020	778 (65%)	164 (54%)	124 (58%)	119 (60%)	1185 (62%)
	2021	801 (70%)	141 (62%)	130 (56%)	112 (56%)	1184 (66%)
	2022	809 (68%)	98 (54%)	150 (51%)	130 (51%)	1187 (62%)
	2023	781 (60%)	110 (54%)	121 (45%)	200 (66%)	1212 (58%)
**Total no. patients discharged to IMC**	2019	1297	147	235	179	1858
	2020	1197	305	212	197	1911
	2021	1142	227	231	199	1799
	2022	1193	181	293	257	1924
	2023	1311	204	270	302	2087
**Total no. bed-blocking days**	2019	4265	670	658	1785	7378
	2020	3060	841	528	1007	5436
	2021	3225	853	602	826	5506
	2022	4896	677	986	1324	7883
	2023	5329	1246	968	3309	10,852
**Total length of stay in hospital (days)**	2019	20,118	2445	3292	4588	30,443
	2020	17,610	4573	2895	3761	28,839
	2021	16,745	2988	2955	3811	26,499
	2022	18,908	2710	4237	5046	30,902
	2023	18,603	3605	3763	7079	33,050
**% of bed-blocking days of total stay in hospital**	2019	21%	27%	20%	39%	24%
	2020	17%	18%	18%	27%	19%
	2021	19%	29%	20%	22%	21%
	2022	26%	25%	23%	26%	26%
	2023	29%	35%	26%	47%	33%
**Average occupied beds due to bed blocking daily**	2019	11.7	1.8	1.8	4.9	20.2
	2020	8.4	2.3	1.4	2.8	14.9
	2021	8.8	2.3	1.6	2.3	15.1
	2022	13.4	1.9	2.7	3.6	21.6
	2023	14.6	3.4	2.7	9.1	29.7
Note. GR = geriatric rehabilitation; STRC = short-term residential care; LTC = long-term care; IMC = intramural care.						

## Appendix G. Transfer Process from Hospital to IMC for Planned Patients

The figure below shows the coordination process for planned patients transferring to GR and part of STRC. The other parts of STRC, hospice, and LTC do not have a planned process. Once it is determined that a patient requires STRC, hospice, or LTC, the coordination process aligns with the emergency process, as the specific type of IMC required is not known prior to hospitalization.

Note. GR = geriatric rehabilitation; STRC = short-term residential care; IMC = intramural care; OR = operating room; EPD = electronic patient dossier

## Appendix H. Transfer Process from Hospital to IMC for Emergency Patients

This process applies to all types of IMC for patients with an emergency hospital admission.

## Appendix I. Number of Cases and Flow Paths per Category, Type of Care, and Type of Hospital Admission

**No.**	**Color in Figure 3**	**No. Distinct Flow Paths**	**No. Cases per Type of Care**				**Total Cases**		**Admission Type**		
			**GR**	**STRC**	**Hospice**	**LTC**	**Total**	**% of total**	**Planned**	**Emergency**	**No Data**
1	Purple	11	78	3	1		82	1%	78	4	
2	Green	22	1119	201	365	217	1902	20%	209	1690	3
3	Purple and green	11	17	3		2	22	0%	16	6	
4	Orange	19	772	167	235	143	1317	14%	120	1194	3
5	Green and orange	58	3868	631	612	742	5853	61%	598	5250	5
6	Purple and orange	18	120	7	1	3	131	1%	122	9	
7	Purple, green and orange	15	30	6	3		39	0%	21	18	
8	No process steps	1	136	46	24	27	233	2%	76	157	
	Total	155	6140	1064	1241	1134	9579	100%	1240	8328	11
Note. GR = geriatric rehabilitation; STRC = short-term residential care; LTC = long-term care.											

## Appendix J. Flow Paths for Group 1 (Purple) and 2 (Green)

**No.**	**Color in** **Figure 3**	**No.** **Distinct** **Flow Paths**	**No. Cases per Type of Care**					**Admission Type**		
			**GR**	**STRC**	**Hospice**	**LTC**	**Total**	**Planned**	**EM**	**No Data**
1	All process steps before hospital admission (purple)	A1-B1-G1-HAD-MT	2				2	2		
		A1-B1-G1-K1-HAE-MT	34	3			37	35	2	
		A1-B1-HAC-MT	1				1		1	
		A1-C1-ZG1-H1-HAE-MT	1				1	1		
		F1-G1-K1-HAE-MT	1				1	1		
		F1-G1-ZC1-D1-HAE-MT	1				1	1		
		F1-ZB1-C1-K1-HAE-MT	1				1	1		
		J1-HAD-MT	19		1		20	19	1	
		J1-K1-HAE-MT	8				8	8		
		J1-ZC1-B1-H1-HAE-MT	1				1	1		
		M1-HAE-MT	9				9	9		
2	All process steps after hospital admission but before medically ready date (green)	HAA-A2-B2-G2-K2-P	333	52	93	73	551	48	502	1
		HAA-A2-B2-G2-Q	35	11	68	10	124	11	113	
		HAA-A2-B2-H2-P	1			1	2	1	1	
		HAA-A2-B2-H2-ZK2-Q	1		1		2	1	1	
		HAA-A2-B2-R	218	85	80	35	418	40	377	1
		HAA-A2-C2-Q	4				4	2	2	
		HAA-A2-C2-ZG2-H2-P	2			1	3	2	1	
		HAA-A2-S	43	13	29	9	94	18	75	1
		HAA-F2-G2-K2-P	231	14	38	54	337	27	310	
		HAA-F2-G2-Q	31	4	16	9	60	6	54	
		HAA-F2-H2-P				2	2	1	1	
		HAA-F2-R	23	3	7	4	37	7	30	
		HAA-F2-ZB2-C2-K2-P	2				2		2	
		HAA-F2-ZB2-C2-Q			2		2		2	
		HAA-J2-K2-P	2				2		2	
		HAA-J2-K2-ZH2-R	1				1	1		
		HAA-J2-Q	187	18	29	18	252	40	212	
		HAA-J2-ZC2-B2-H2-P				1	1		1	
		HAA-J2-ZC2-B2-R			2		2		2	
		HAA-J2-ZC2-S		1			1		1	
		HAA-M2-P	4				4	4		
		HAA-M2-ZH2-G2-Q	1				1		1	
Note. GR = geriatric rehabilitation; STRC = short-term residential care; LTC = long-term care; EM = emergency.										

## Appendix K. Flow Paths for Group 3 (Purple and Green) and 4 (Orange)

**No.**	**Color in** **Figure 3**	**No. Distinct** **Flow Paths**	**No. Cases per Type of Care**					**Admission Type**		
			**GR**	**STRC**	**Hospice**	**LTC**	**Total**	**Planned**	**EM**	**No Data**
3	All process steps partly before and partly after hospital admission but before medically ready date (purple and green)	A1-B1-G1-HAD-M2-P	8			1	9	9		
		A1-B1-H1-HAE-J2-Q	1				1	1		
		A1-B1-HAC-M2-P	1				1	1		
		A1-C1-HAD-F2-H2-P	1				1	1		
		A1-HAB-F2-G2-K2-P	1	1		1	3	1	2	
		F1-HAC-A2-S	1				1		1	
		F1-HAC-J2-K2-P	1				1	1		
		J1-HAD-A2-B2-H2-P	1				1	1		
		J1-HAD-F2-H2-P		1			1	1		
		M1-HAE-A2-B2-G2-Q	1	1			2		2	
		M1-HAE-F2-G2-Q	1				1		1	
4	All process steps after medically ready date (orange)	HAA-MRA-A3-B3-G3-K3-N3	380	96	129	75	680	53	625	2
		HAA-MRA-A3-B3-G3-L3	26	11	37	6	80	12	68	
		HAA-MRA-A3-B3-H3-N3	1				1		1	
		HAA-MRA-A3-B3-H3-ZK3-L3	2				2		2	
		HAA-MRA-A3-B3-I3	60	17	22	17	116	15	101	
		HAA-MRA-A3-C3-K3-N3	3				3	1	2	
		HAA-MRA-A3-C3-L3	2				2		2	
		HAA-MRA-A3-C3-ZG3-H3-N3			1		1		1	
		HAA-MRA-A3-C3-ZG3-I3		1			1		1	
		HAA-MRA-A3-E3	11	9	3	4	27		27	
		HAA-MRA-F3-G3-K3-N3	182	9	28	17	236	16	219	1
		HAA-MRA-F3-G3-L3	22	1	10	6	39	4	35	
		HAA-MRA-F3-G3-ZC3-D3-N3	2				2		2	
		HAA-MRA-F3-I3	3	1	2	2	8		8	
		HAA-MRA-F3-ZB3-E3	1				1		1	
		HAA-MRA-J3-L3	24	1	1		26	4	22	
		HAA-MRA-J3-ZG3-H3-N3	1			1	2		2	
		HAA-MRA-M3-N3	1			1	2	1	1	
		HAA-MRA-O	51	21	2	14	88	14	74	
Note. GR = geriatric rehabilitation; STRC = short-term residential care; LTC = long-term care; EM = emergency.										

## Appendix L. Flow Paths for Group 5 (Green and Orange), Part 1

**No.**	**Color in** **Figure 3**	**No.** **Distinct** **Flow Paths**	**No. Cases per Type of Care**					**Admission Type**			
			**GR**	**STRC**	**Hospice**	**LTC**	**Total**	**Planned**	**EM**	**No Data**	
5	All process steps after hospital admission, partly before and partly after medically ready date (green and orange)	HAA-A2-B2-G2-K2-MRE-O	1078	163	125	204	1570	170	1400		
		HAA-A2-B2-G2-MRD-M3-N3	1106	198	198	138	1640	143	1495		2
		HAA-A2-B2-G2-MRD-O	37	12	29	10	88	7	81		
		HAA-A2-B2-H2-MRE-J3-L3	1	1		2	4		4		
		HAA-A2-B2-H2-ZK2-MRD-O	4			1	5		5		
		HAA-A2-B2-MRC-J3-K3-N3	132	39	44	47	262	24	237		1
		HAA-A2-B2-MRC-J3-L3	10	8	7	3	28	2	26		
		HAA-A2-B2-MRC-M3-ZK3-L3		1			1		1		
		HAA-A2-B2-MRC-O	67	28	7	17	119	16	103		
		HAA-A2-C2-K2-MRE-F3-I3	1	1			2		2		
		HAA-A2-C2-K2-MRE-O	1			1	2	1	1		
		HAA-A2-C2-MRD-F3-H3-N3	9	2	4	2	17		17		
		HAA-A2-C2-MRD-F3-I3				1	1		1		
		HAA-A2-C2-MRD-M3-ZH3-I3			1		1		1		
		HAA-A2-C2-MRD-O	2				2		2		
		HAA-A2-C2-ZG2-H2-MRE-O	5			1	6	1	5		
		HAA-A2-C2-ZG2-MRC-M3-N3	1		1	1	3	2	1		
		HAA-A2-D2-MRE-F3-G3-L3			1		1		1		
		HAA-A2-D2-ZH2-G2-MRD-O	1				1		1		
		HAA-A2-MRB-F3-G3-K3-N3	568	80	83	82	813	86	726		1
		HAA-A2-MRB-F3-G3-L3	28	4	20	7	59	4	55		
		HAA-A2-MRB-F3-H3-ZK3-L3	1				1		1		
		HAA-A2-MRB-F3-I3	100	41	18	20	179	22	157		
Note. GR = geriatric rehabilitation; STRC = short-term residential care; LTC = long-term care; EM = emergency.										

## Appendix M. Flow Paths for Group 5 (Green and Orange), Part 2

**No.**	**Color in** **Figure 3**	**No.** **Distinct** **Flow Paths**	**No. Cases per Type of Care**					**Admission Type**		
			**GR**	**STRC**	**Hospice**	**LTC**	**Total**	**Planned**	**EM**	**No Data**
5	All process steps after hospital admission, partly before and partly after medically ready date (green and orange)	HAA-A2-MRB-J3-K3-ZH3-I3		1			1		1	
		HAA-A2-MRB-J3-L3	2				2	1	1	
		HAA-A2-MRB-J3-ZG3-H3-N3	2	1			3	1	2	
		HAA-A2-MRB-M3-N3	1				1		1	
		HAA-A2-MRB-O	46	8	14	16	84	7	77	
		HAA-F2-G2-K2-MRE-A3-E3	1				1		1	
		HAA-F2-G2-K2-MRE-O	96	11	6	27	140	15	125	
		HAA-F2-G2-MRD-A3-D3-N3	1				1		1	
		HAA-F2-G2-MRD-M3-N3	96	4	11	36	147	18	129	
		HAA-F2-G2-MRD-O	17		2	10	29	3	26	
		HAA-F2-G2-ZC2-D2-MRE-O			1		1		1	
		HAA-F2-G2-ZC2-MRB-M3-N3	1				1		1	
		HAA-F2-H2-MRE-O	2				2	1	1	
		HAA-F2-MRC-A3-C3-K3-N3	1		2	2	5		5	
		HAA-F2-MRC-J3-K3-N3	321	14	22	92	449	40	408	1
		HAA-F2-MRC-J3-L3	40		6	8	54	10	44	
		HAA-F2-MRC-M3-N3	1				1		1	
		HAA-F2-MRC-O	14	2	1	3	20	2	18	
		HAA-F2-ZB2-C2-K2-MRE-O	5	3	1	4	13		13	
		HAA-F2-ZB2-C2-MRD-M3-N3	6	1	3	1	11	2	9	
		HAA-F2-ZB2-MRB-J3-K3-N3	1		3		4	1	3	
		HAA-F2-ZB2-MRB-O	2				2		2	
		HAA-J2-K2-MRE-A2-B3-I3	1				1		1	
		HAA-J2-K2-MRE-O	2				2	1	1	
		HAA-J2-MRD-A3-B3-H3-N3		1			1		1	
		HAA-J2-MRD-M3-N3				1	1		1	
		HAA-J2-MRD-O	48	4		5	57	16	41	
		HAA-J2-ZC2-B2-H2-MRE-O			1		1		1	
		HAA-J2-ZC2-B2-MRC-M3-N3	1	1	1		3		3	
		HAA-M2-MRE-F3-G3-L3	1				1	1		
		HAA-M2-MRE-O	1				1	1		
		HAA-M2-ZD2-B2-G2-MRD-O	5				5		5	
		HAA-M2-ZD2-MRB-F3-G3-L3		1			1		1	
		HAA-M2-ZH2-G2-MRD-O		1			1		1	
		HAA-M2-ZH2-G2-ZC2-MRB-O	1				1		1	
Note. GR = geriatric rehabilitation; STRC = short-term residential care; LTC = long-term care; EM = emergency.										

## Appendix N. Flow Paths for Group 6 (Purple and Orange)

**No.**	**Color in Figure 3**	**No. Distinct Flow Paths**	**No. Cases per Type of Care**					**Admission Type**		
			**GR**	**STRC**	**Hospice**	**LTC**	**Total**	**Planned**	**EM**	**No Data**
6	All process steps partly before hospital admission and partly after medically ready date (purple and orange)	A1-B1-G1-HAD-MRA-M3-N3		1			1	1		
		A1-B1-G1-HAD-MRA-O	4				4	4		
		A1-B1-G1-K1-HAE-MRA-O	87	6			93	93		
		A1-B1-HAC-MRA-O	2				2	2		
		A1-C1-ZG1-H1-HAE-MRA-O	4				4	4		
		A1-D1-HAE-MRA-O	1				1	1		
		A1-HAB-MRA-F3-G3-K3-N3	4		1	1	6		6	
		F1-G1-K1-HAE-MRA-A3-E3	1				1	1		
		F1-G1-K1-HAE-MRA-O	1				1	1		
		F1-HAC-MRA-J3-K3-N3	1			2	3	1	2	
		F1-ZB1-C1-K1-HAE-MRA-O	2				2	2		
		J1-HAD-MRA-F3-H3-N3	1				1	1		
		J1-HAD-MRA-O	1				1	1		
		J1-K1-HAE-MRA-A3-B3-I3	1				1	1		
		J1-K1-HAE-MRA-O	5				5	5		
		J1-ZC1-B1-H1-HAE-MRA-O	3				3	3		
		J1-ZC1-B1-HAC-MRA-O	1				1	1		
		M1-HAE-MRA-F3-G3-L3	1				1		1	
Note. GR = geriatric rehabilitation; STRC = short-term residential care; LTC = long-term care; EM = emergency.										

## Appendix O. Flow Paths for Group 7 (Purple, Green, and Orange) and 8 (No Process Steps)

**No.**	**Color in Figure 3**	**No. Distinct Flow Paths**	**No. Cases per Type of Care**					**Admission Type**		
			**GR**	**STRC**	**Hospice**	**LTC**	**Total**	**Planned**	**EM**	**No Data**
7	All process steps partly before and partly after hospital admission but before and partly after medically ready date (purple, green, and orange)	A1-B1-G1-HAD-M2-MRE-O	10	2			12	12		
		A1-B1-HAC-J2-MRD-M3-N3	1				1	1		
		A1-C1-HAD-F2-H2-MRE-O	1				1	1		
		A1-HAB-F2-G2-K2-MRE-O	4	2			6	4	2	
		A1-HAB-F2-G2-MRD-M3-N3	2	1			3		3	
		A1-HAB-F2-MRC-J3-K3-N3	1		1		2		2	
		A1-HAB-F2-MRC-O		1			1		1	
		F1-HAC-A2-C2-MRD-M3-N3	1		1		2		2	
		F1-HAC-A2-MRB-O	1				1		1	
		J1-HAD-A2-B2-H2-MRE-O	2				2	1	1	
		J1-HAD-A2-MRB-F3-H3-N3	1				1		1	
		J1-K1-HAE-A2-B2-MRC-O	1				1	1		
		M1-HAE-A2-B2-G2-MRD-O	2		1		3		3	
		M1-HAE-F2-MRC-J3-L3	2				2		2	
		M1-ZK1-HAD-A2-B2-MRC-O	1				1	1		
8	No process steps	HAA-MT	136	46	24	27	233	76	157	
Note. GR = geriatric rehabilitation; STRC = short-term residential care; LTC = long-term care; EM = emergency.										

## Appendix P. Time Between Coordination Steps from Hospital to IMC

**Time in Days Between:**		**GR**	**STRC**	**Hospice**	**LTC**	**Total**	**Total Planned**	**Total Emergency**
Hospital admission (step 1) and medically ready (step 2)	M	11.0	10.8	10.4	13.1	11.2	10.8	11.1
	Mdn	8.0	8.0	8.0	10.0	8.0	7.0	8.0
	SD	11.7	10.2	9.2	11.6	11.3	11.5	10.7
Medically ready (step 2) and order to transfer (step 3)	M	−4.9	−4.6	−2.8	−7.9	−4.9	−7.9	−4.5
	Mdn	−3.0	−3.0	−2.0	−5.0	−3.0	−4.0	−3.0
	SD	7.4	6.7	5.0	9.4	7.4	13.5	6.0
Order to transfer (step 3) and open order (step 4)	M	2.8	2.5	1.5	3.2	2.6	2.0	2.6
	Mdn	2.0	1.0	1.0	2.0	2.0	1.0	2.0
	SD	4.7	3.6	2.8	3.8	4.3	4.4	3.5
Open order (step 4) and registration at IMC facility (step 5)	M	1.4	1.6	1.5	3.7	1.5	1.2	1.8
	Mdn	0.0	0.0	0.0	1.0	0.0	0.0	0.0
	SD	4.3	4.3	4.4	5.9	4.4	7.3	4.1
Registration at IMC facility (step 5) and confirmation of IMC facility (step 6)	M	2.3	3.3	2.6	6.2	2.9	4.6	2.8
	Mdn	1.0	1.0	1.0	3.0	1.0	1.0	1.0
	SD	4.8	6.4	4.4	8.9	5.7	11.9	5.3
Confirmation of IMC facility (step 6) and hospital discharge (step 7)	M	3.2	2.9	1.8	4.7	3.2	4.7	3.0
	Mdn	2.0	2.0	1.0	3.1	2.0	3.0	2.0
	SD	3.4	4.3	2.7	5.1	3.7	5.7	3.3
Note. M = mean; Mdn = median; SD = standard deviation; GR = geriatric rehabilitation; STRC = short-term residential care; LTC = long-term care.								

## Appendix Q. Time Between Coordination Steps from Hospital to IMC Types, Planned vs. Emergency Hospital Admission

**Time in Days Between:**		**GR Planned**	**GR** **Emergency**	**STRC Planned**	**STRC Emergency**	**Hospice Planned**	**Hospice Emergency**	**LTC Planned**	**LTC Emergency**
Hospital admission (step 1) and medically ready (step 2)	M	10.6	10.9	8.1	11.1	12.1	10.2	15.0	13.0
	Mdn	7.0	8.0	4,0	8.0	11.0	8.0	10.5	10.0
	SD	11.8	10.9	9.5	10.3	8.7	9.2	13.7	11.4
Medically ready (step 2) and order to transfer (step 3)	M	−9.1	−4.2	−5.8	−4.5	−2.6	−2.9	−6.9	−7.9
	Mdn	−4.0	−3.0	−2.0	−3.0	−2.0	−2.0	−5.0	−6.0
	SD	14.8	5.0	9.5	6.3	3.6	5.1	7.5	9.4
Order to transfer (step 3) and open order (step 4)	M	2.0	2.8	2.2	2.5	1.3	1.6	2.3	3.2
	Mdn	1.0	2.0	1.0	1.0	1.0	1.0	1.0	2.0
	SD	4.9	3.5	3.0	3.6	2.1	2.8	2.7	3.9
Open order (step 4) and registration at IMC facility (step 5)	M	1.0	1.4	0.9	1.7	1.6	1.5	3.9	3.7
	Mdn	0.0	0.0	0.0	0.0	0.0	0.0	1.0	1.0
	SD	8.1	3.4	3.2	4.5	3.6	4.5	6.0	5.9
Registration at IMC facility (step 5) and confirmation of IMC facility (step 6)	M	4.8	2.0	3.4	3.4	2.8	2.6	6.4	6.3
	Mdn	1.0	1.0	1.0	1.0	1.0	1.0	4.0	3.0
	SD	13.1	3.5	6.9	6.9	4.9	4.3	7.2	9.2
Confirmation of IMC facility (step 6) and hospital discharge (step 7)	M	5.1	2.9	3.4	2.8	1.4	1.9	6.1	4.6
	Mdn	3.0	2.0	2.0	2.0	1.0	1.0	4.0	3.0
	SD	5.9	2.7	4.5	4.2	1.7	2.8	6.3	5.0
Note. M = mean; Mdn = median; SD = standard deviation; GR = geriatric rehabilitation; STRC = short-term residential care; LTC = long-term care.									
